# Residential mobility and liver cancer risk: findings from a prospective cohort study in Chinese women

**DOI:** 10.1186/s12889-024-18574-y

**Published:** 2024-04-29

**Authors:** Jia-Yi Tuo, Qiu-Ming Shen, Zhuo-Ying Li, Jing-Yu Tan, Jie Fang, Li-Feng Gao, Yu-Ting Tan, Hong-Lan Li, Yong-Bing Xiang

**Affiliations:** 1 State Key Laboratory of Systems Medicine for Cancer, Shanghai Cancer Institute, Renji Hospital, Shanghai Jiao Tong University School of Medicine, No. 25, Lane 2200, Xie Tu Road, 200032 Shanghai, P. R. China; 2 School of Public Health, Shanghai Jiao Tong University School of Medicine, 200025 Shanghai, P. R. China; 3 School of Public Health, Fudan University, 200032 Shanghai, P. R. China

**Keywords:** Liver cancer, Residential mobility, Migration, Cohort study

## Abstract

**Background:**

Residential mobility is believed to influence the occurrence and development of cancer; however, the results are inconclusive. Furthermore, limited studies have been conducted on Asian populations. This study aimed to evaluate the relationship between residential mobility and liver cancer risk among Chinese women.

**Methods:**

We enrolled 72,818 women from urban Shanghai between 1996 and 2000, and then followed them until the end of 2016. Cox regression models were used to calculate hazard ratios (HRs) and 95% confidence intervals (CIs) to assess the association between residential mobility and liver cancer risk. A linear trend test was conducted by ranking variables. A sensitivity analysis was also conducted, excluding participants with follow-up times of less than 2 years, to prevent potential bias.

**Results:**

During the 1,269,765 person-years of follow-up, liver cancer was newly diagnosed in 259 patients. Domestic migration (HR = 1.47, 95% CI, 1.44–1.50), especially immigration to Shanghai (HR = 1.47, 95% CI, 1.44–1.50) was associated with an increased risk of liver cancer. In addition, migration frequency, age at initial migration and first immigration to Shanghai had linear trends with an increased liver cancer risk (*P*_*trend*_ <0.001). The results were similar when excluding participants with less than two years of follow-up.

**Conclusions:**

The possible association between residential mobility and a higher risk of liver cancer in women could suggest the need for effective interventions to reduce adverse environmental exposures and enhance people’s health.

## Background

Primary liver cancer ranks sixth as the most common cancer, and the third leading cause of cancer-related death all over the world in 2020 [1]. China accounts for half of this. In 2015, 466,000 newly diagnosed cases of liver cancer and 422,000 cancer-related deaths were reported in China [2]. The high occurrence and mortality rates of liver cancer highlight the urgent need for optimal strategies for treatment and prevention in high-risk populations. Currently, most risk factors for liver cancer have been reasonably defined. Aflatoxin contamination, as well as hepatitis B (HBV) and C (HCV) virus infections, are attributed to nearly 80% of liver cancer cases [3–5]. In addition, unhealthy dietary patterns, poor lifestyle habits, and chronic diseases are well-known risk factors for liver cancer [4, 6, 7]. However, the known risk factors do not account for all the causes of liver cancer, and there may be additional significant risk factors that could contribute to cancer occurrence.

Residential mobility is important in human society. In the past decades, the scale of domestic and international migration has increased sharply [8], and China has experienced large-scale migration from the last century until now [9]. The studies focusing on the effects of residential mobility on health, including cancer occurrence, have been growing. However, the results are still inconclusive [10–13]. A case-control study observed that adults with a history of cancer had a higher proportion of residential mobility [12], while no elevated risk was found between higher residential mobility and childhood leukemia occurrence [11]. However, no studies have explored the association between residential mobility and liver cancer risk, particularly among the areas with higher liver cancer incidence like China. In addition, many studies found the pathways residential mobility might affect cancer occurrence including (1) exposure to adverse environmental factors; (2) mental disorders because of family separation and less social support; (3) more likely to choose long-time work and face heavy working stress in unfavorable working environments; (4) new environmental adaption stress; (5) higher levels of behavioral problems and lack of healthcare [8, 14, 15]. More evidence is needed to explore the association of residential mobility on cancer occurrence, especially on liver cancer risk.

Most previous epidemiologic studies investigating cancer etiology were conducted in North American and Western European countries that are relatively homogenous in terms of cancer spectrums and many lifestyle exposures. However, China has become an increasing country of cancer burden and has different risk factors from other countries. A large prospective cohort study in China is important to evaluate cancer development. Shanghai has been one of the largest immigration cities in the mainland of China over the past few decades, with diverse cultures and a prosperous economy. In this study, we used data from the Shanghai Women’s Health Study, a population-based prospective cohort study in China, which provided thorough information on crucial variables, especially on residential history, in order to find reliable evidence regarding the association of residential mobility on liver cancer risk among Chinese women, which could help prevent liver cancer and promote public health.

## Materials and methods

### Study population

**Fig. 1:**
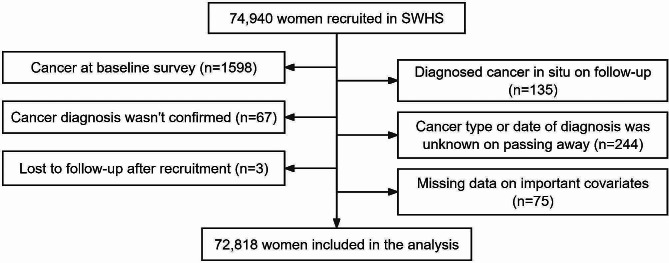
Screening process in the SWHS

In our study, we recruited 74,940 women aged 40 to 70 years who lived in urban Shanghai from December 1996 to May 2000 in the Shanghai Women’s Health Study (SWHS). A previously published report provides a detailed study design and rationale [16]. Each participant was interviewed and asked to complete a questionnaire that included basic demographic data, residential histories, lifestyle preferences, physical activity, smoking status, alcohol drinking status, tea drinking status, personal medical history of chronic diseases, and family history of cancer. When measuring body index, we mainly collected weight and height data to get the exact body mass index (BMI) value. A calibrated weight scale was used to measure body weight, and a tape measure was used to measure height. Participants were asked to take off their clothes and shoes and stand upright when their height was measured. In this study, participants who met the following criteria were excluded: (1) had cancer at baseline survey (*n* = 1,598); (2) diagnosed with cancer in situ during follow-up (*n* = 135); (3) cancer diagnosis was not confirmed (*n* = 67); (4) cancer type or date of diagnosis was unknown on passing away (*n* = 244); (5) lost to follow-up after recruitment (*n* = 3); and (6) had missing data on important covariates, including BMI, education, family income, family history of liver cancer, residential address, and residential year (*n* = 75). A total of 72,818 participants were included in this study. The screening process of our participants in the SWHS has been presented (Fig. 1). The cohort protocol has been approved by the Institutional Review Boards of the Shanghai Cancer Institute and Vanderbilt University. Informed consent has been obtained from all participants. The current study has been approved by the Renji Hospital Ethics Committee of Shanghai Jiao Tong University School of Medicine (KY2019-197).

### Exposures and covariates

The data about residential mobility in the past twenty years was collected only at baseline survey, including domestic migration status (defined as “ever/never left the place of residence across city boundaries more than one year”, yes/no), domestic migration frequency (3 categories: not migrated, once, twice or more times), age at initial domestic migration (5 categories: not migrated, under 18 years old, 18–24 years old, 25–34 years old, 35 years old and above), immigration to Shanghai status (defined as “ever/never migrated from other cities to Shanghai”, yes/no), age at first immigration to Shanghai (5 categories: not migrated, under 18 years old, 18–24 years old, 25–34 years old, 35 years old and above).

These variables have been chosen as main covariates: age at entry (continuous), menopausal status (yes/no), education (4 categories: elementary school and below, middle school, high school, college and above), marital status (living with spouse/living without spouse), number of children (5 categories: 0, 1, 2, 3–4, ≥ 5), physical activity (multiplying the weekly hours spent on specific physical activities in the past 1 year by their corresponding MET values and cumulating the total weekly MET, metabolic equivalent (MET) h/week, continuous), BMI (kg/m^2^, continuous), calorie intake (kcal/day, continuous), family income (Yuan per year, 4 categories: <10,000, 10,000–19,999, 20,000–29,999 and ≥ 30,000), occupation (4 categories: housewife, professional, clerical, manual workers), family history of liver cancer (yes/no), tea drinking (defined as “ever drank tea at least 3 times/week for more than 6 months continuously”, yes/no), alcohol drinking (defined as “ever drank alcohol at least 3 times/week for more than 6 months continuously”, yes/no), smoking (defined as “ever smoked at least 1 cigarette/day for more than 6 months continuously”, yes/no), medical history of hypertension (yes/no), type 2 diabetes (yes/no), cholelithiasis (yes/no) and hepatitis (yes/no).

### Follow-up and case ascertainment

Through every 3–4 years of follow-up surveys, we tracked all the participants until they had cancer. We linked records annually with the databases of the Shanghai Resident Registry, Shanghai Vital Statistics Registry, and Shanghai Cancer Registry [17]. The response rates of the five follow-up surveys were 99.7% (2000–2002), 98.7% (2002–2004), 94.9% (2004–2006), 92.3% (2007–2010), and 91.1% (2012–2017). All new cases of liver cancer were confirmed by home visits, medical records from hospitals, and medical charts reviewed by professors. Furthermore, the International Classification of Disease, Ninth Revision (ICD-9) was used to code all of the cancers, and liver cancer was classified with the code 155 [18]. The follow-up information of our study was censored on 31 December 2016.

### Statistical analyses

All the participants were divided into non-cases and liver cancer cases and were also compared by domestic migration experience. We used medians with interquartile ranges to describe continuous variables and presented categorical variables using counts and proportions. We used the Wilcoxon-Mann-Whitney test to compare continuous variables because of their skewed distribution and the χ^2^ test to compare categorical variables.

We used Cox proportional regression models to explore the relationship between residential mobility and the incidence of liver cancer [19]. We used the time from baseline to outcome (i.e., liver cancer occurrence) or right-censoring (e.g. death, loss to follow-up, or December 31, 2016), which came first, to calculate the person-years (PYs). The underlying time metric in the Cox model was the follow-up period. In addition, we used the residual method to check the assumptions of proportional hazards for residential history, and no evidence was detected of the violation of the assumption. We obtained hazard ratios (HRs) and 95% confidence intervals (CIs) from three models: the unadjusted model (Model 1), the model adjusted for the selected covariates (age at entry, menopausal status, education, family income, occupation, marital status, number of children, Model 2), and the model further adjusted for other selected covariates (BMI, physical activity, calorie intake, smoking, alcohol drinking, tea drinking, medical history of diabetes, hypertension, cholelithiasis, hepatitis and family history of liver cancer, Model 3). A linear trend test was performed by recoding categorical variables as rank variables. We also conducted sensitivity analyses by excluding participants whose follow-up times were less than 2 years, to avoid the potential bias in the cohort studies.

The results were considered statistically significant if the two-sided *P*-values were < 0.05. Cox proportional hazards regression models were performed by using the ‘survival’ package in R software (version 4.0.5).

## Results

Between the baseline survey time and the end of 2016, we observed a total of 259 new cases of primary liver cancer among female participants. The total follow-up times were approximately 1.27 million PYs (average = 17.44 years). The density incidence of liver cancer was 20.40 per 100,000 PYs, and the cumulative incidence was 0.36% during the follow-up period. Table 1 shows that compared with non-cancer participants, those with liver cancer were relatively older, menopausal, fatter, and more likely to have a medical history of type 2 diabetes, cholelithiasis, hepatitis, hypertension, and family history of liver cancer. Women who never drank tea were more likely to have liver cancer. Additionally, participants with liver cancer had a higher proportion of lacking education, poverty, living without a spouse, and more children than non-cases. The results of these variables were similar when we compared women who ever and never migrate.

**Table 1:** Baseline demographic characteristics and selected variables of the study population (SWHS, 1996–2016)

	Overall (*n* = 72,818)		*P*	Overall (*n* = 72,818)		*P*
	Liver cancer cases (*n* = 259)	Non-cases (*n* = 72,559)		Ever migrated (*n* = 9,086)	Never migrated (*n* = 63,732)	
Age at entry (years)	60.8 (14.6)	50.2 (16.4)	< 0.001	50.7 (15.7)	50.2 (16.6)	< 0.001
Menopausal status (yes)	198 (76.4)	35,360 (48.7)	< 0.001	4,699 (51.7)	30,859 (48.4)	< 0.001
Education (*n*, %)			< 0.001			< 0.001
Elementary school and below	109 (42.1)	15,419 (21.3)		2,272 (25.0)	13,256 (20.8)	
Middle school	65 (25.1)	27,044 (37.3)		3,293 (36.2)	23,816 (37.4)	
High school	61 (23.6)	20,296 (28.0)		2,057 (22.6)	18,300 (28.7)	
College and above	24 (9.3)	9,800 (13.5)		1,464 (16.1)	8,360 (13.1)	
Family income (Yuan/year)			0.001			< 0.001
< 10,000	60 (23.2)	11,660 (16.1)		1,752 (19.3)	9,968 (15.6)	
10,000-19,999	95 (36.7)	27,744 (38.2)		3,439 (37.8)	24,400 (38.3)	
20,000-29,999	77 (29.7)	20,391 (28.1)		2,351 (25.9)	18,117 (28.4)	
≥ 30,000	27 (10.4)	12,764 (17.6)		1,544 (17.0)	11,247 (17.6)	
Occupation (*n*, %)			0.031			< 0.001
Housewife	3 (1.2)	265 (0.4)		56 (0.6)	212 (0.3)	
Professional	58 (22.4)	20,640 (28.4)		2,875 (31.6)	17,823 (28.0)	
Clerical	59 (22.8)	15,061 (20.8)		1,755 (19.3)	13,365 (21.0)	
Manual workers	139 (53.7)	36,593 (50.4)		4,400 (48.4)	32,332 (50.7)	
Marital status (*n*, %)			0.267			< 0.001
Living with spouse	224 (86.5)	64,472 (88.9)		7,701 (84.8)	56,995 (89.4)	
Living without spouse	35 (13.5)	8,087 (11.1)		1,385 (15.2)	6,737 (10.6)	
Number of children (*n*, %)			< 0.001			< 0.001
0	7 (2.7)	2,395 (3.3)		365 (4.0)	2,037 (3.2)	
1	86 (33.2)	40,348 (55.6)		4,475 (49.3)	35,959 (56.4)	
2	59 (22.8)	15,447 (21.3)		2,102 (23.1)	13,404 (21.0)	
3–4	89 (34.4)	12,001 (16.5)		1,760 (19.4)	10,330 (16.2)	
≥5	18 (6.9)	2,368 (3.3)		384 (4.2)	2,002 (3.1)	
BMI (kg/m^2^)	24.7 (5.4)	23.7 (4.4)	< 0.001	24.2 (3.5)	24.0 (3.4)	< 0.001
Physical activity (MET-hours/week)	102.6 (55.3)	100.5 (57.1)	0.980	100.3 (57.3)	101.5 (56.3)	0.023
Calorie intake (kcal/day)	1,587.2 (474.7)	1,634.8 (495.4)	0.133	1,634.0 (494.9)	1,636.9 (499.2)	0.885
Smoking status (yes)	11 (4.2)	2,007 (2.8)	0.208	324 (3.6)	1,694 (2.7)	< 0.001
Alcohol drinking status (yes)	4 (1.5)	1,636 (2.3)	0.576	203 (2.2)	1,437 (2.3)	0.932
Tea drinking status (yes)	58 (22.4)	21,723 (29.9)	0.010	2,664 (29.3)	19,117 (30.0)	0.192
Family history of liver cancer (yes)	26 (10.0)	2,365 (3.3)	< 0.001	274 (3.0)	2,117 (3.3)	0.134
History of hepatitis (yes)	37 (14.3)	1,836 (2.5)	< 0.001	236 (2.6)	1,637 (2.6)	0.899
History of cholelithiasis (yes)	52 (20.1)	8,053 (11.1)	< 0.001	975 (10.7)	7,130 (11.2)	0.202
History of diabetes (yes)	26 (10.0)	3,106 (4.3)	< 0.001	415 (4.6)	2,717 (4.3)	0.190
History of hypertension (yes)	77 (29.7)	17,129 (23.6)	0.025	2,245 (24.7)	14,961 (23.5)	0.010

SWHS, Shanghai Women’s Health Study; BMI, body mass index; MET, Metabolic equivalent

Values were shown in median (interquantile range) for continuous variables and count (proportion) for categorical items

Baseline information about residential mobility is shown in Table 2. Women with liver cancer were more likely to have domestic migration especially immigration to Shanghai than those without liver cancer. In addition, liver cancer cases have a higher proportion of migration frequency, older at initial migration and first immigration to Shanghai than non-cases. However, no significant difference was observed among these interesting factors.

**Table 2:** Residential mobility of the study population (SWHS, 1996–2016)

	Overall (*n* = 72,818)	Liver cancer cases (*n* = 259)	Non-cases (*n* = 72,559)	*P*
Migration status (*n*, %)				0.055
Yes	9,086 (12.5)	43 (16.6)	9,043 (12.5)	
No	63,732 (87.5)	216 (83.4)	63,516 (87.5)	
Migration frequency (*n*, %)				0.066
No migration	63,732 (87.5)	216 (83.4)	63,516 (87.5)	
Once	6,548 (9.0)	34 (13.1)	6,514 (9.0)	
Twice or more times	2,538 (3.5)	9 (3.5)	2,529 (3.5)	
Age at initial migration (*n*, %)				0.370
No migration	63,732 (87.5)	216 (83.4)	63,516 (87.5)	
Under 18 years old	2,107 (2.9)	10 (3.9)	2,097 (2.9)	
18–24 years old	2,274 (3.1)	11 (4.2)	2,263 (3.1)	
25–34 years old	2,766 (3.8)	12 (4.6)	2,754 (3.8)	
35 years old and above	1,939 (2.7)	10 (3.9)	1,929 (2.7)	
Immigration to Shanghai status (*n*, %)				0.052
Yes	9,058 (12.4)	43 (16.6)	9,015 (12.4)	
No	63,760 (87.6)	216 (83.4)	63,544 (87.6)	
Age at first immigration to Shanghai (*n*, %)				0.209
No immigration	63,760 (87.6)	216 (83.4)	63,544 (87.6)	
Under 18 years old	1,282 (1.8)	7 (2.7)	1,275 (1.8)	
18–24 years old	1,670 (2.3)	8 (3.1)	1,662 (2.3)	
25–34 years old	3,020 (4.1)	11 (4.2)	3,009 (4.1)	
35 years old and above	3,086 (4.2)	17 (6.6)	3,069 (4.2)	

HRs and 95% CIs of liver cancer for residential mobility were presented in Table 3, including domestic migration status, migration frequency, age at initial migration, immigration to Shanghai status, and age at first immigration to Shanghai among the cohort. All three models suggested that domestic migration (HR = 1.44, 95% CI, 1.41–1.48; HR = 1.47, 95% CI, 1.44–1.50; HR = 1.47, 95% CI, 1.44–1.50), and immigration to Shanghai (HR = 1.44, 95% CI, 1.41–1.47; HR = 1.47, 95% CI, 1.44–1.50; HR = 1.47, 95% CI, 1.44–1.50) were associated with increased risk of liver cancer. In addition, migration frequency, age at initial migration, and first immigration to Shanghai all had linear trends with significantly increased liver cancer risk in Model 1–3 (*P*_*trend*_ <0.001).

**Table 3:** HRs and 95% CIs of liver cancer for residential mobility among the study population (SWHS, 1996–2016)

	Cases (*n*)	Total (*n*)	PYs (years)	HR (95% CI)^1^	HR (95% CI)^2^	HR (95% CI)^3^
Migration status						
No	216	63,732	1,114,065.7	1 (reference)	1 (reference)	1 (reference)
Yes	43	9,086	155,699.1	1.44 (1.41, 1.48)	1.47 (1.44, 1.50)	1.47 (1.44, 1.50)
Migration frequency						
No migration	216	63,732	1,114,065.7	1 (reference)	1 (reference)	1 (reference)
Once	34	6,548	111,824.3	1.49 (1.45, 1.53)	1.51 (1.47, 1.55)	1.51 (1.47, 1.55)
Twice or more times	9	2,538	43,874.9	1.34 (1.29, 1.40)	1.37 (1.32, 1.43)	1.37 (1.32, 1.43)
*P* for trend				< 0.001	< 0.001	< 0.001
Age at initial migration						
No migration	216	63,732	1,114,065.7	1 (reference)	1 (reference)	1 (reference)
Under 18 years old	10	2,107	35,094.9	1.85 (1.77, 1.93)	1.76 (1.69, 1.84)	1.75 (1.68, 1.83)
18–24 years old	11	2,274	38,164.8	1.69 (1.62, 1.76)	1.63 (1.56, 1.70)	1.62 (1.56, 1.69)
25–34 years old	12	2,766	48,619.4	1.29 (1.24, 1.34)	1.36 (1.31, 1.41)	1.36 (1.31, 1.42)
35 years old and above	10	1,939	33,820.1	1.17 (1.12, 1.23)	1.25 (1.19, 1.31)	1.26 (1.20, 1.32)
*P* for trend				< 0.001	< 0.001	< 0.001
Immigration to Shanghai status						
No	216	63,760	1,114,558.9	1 (reference)	1 (reference)	1 (reference)
Yes	43	9,058	155,205.9	1.44 (1.41, 1.47)	1.47 (1.44, 1.50)	1.47 (1.44, 1.50)
Age at first immigration to Shanghai						
No immigration	216	63,760	1,114,558.9	1 (reference)	1 (reference)	1 (reference)
Under 18 years old	7	1,282	21,014.1	2.28 (2.15, 2.41)	2.06 (1.95, 2.18)	2.05 (1.94, 2.17)
18–24 years old	8	1,670	27,004.2	2.17 (2.07, 2.28)	1.93 (1.84, 2.03)	1.91 (1.82, 2.01)
25–34 years old	11	3,020	53,145.4	1.33 (1.28, 1.37)	1.39 (1.34, 1.45)	1.39 (1.34, 1.44)
35 years old and above	17	3,086	54,042.2	1.16 (1.12, 1.21)	1.23 (1.19, 1.28)	1.24 (1.20, 1.29)
*P* for trend				< 0.001	< 0.001	< 0.001

Model 1 was unadjusted;

Model 2 was adjusted for age at entry, menopausal status, education, family income, occupation, marital status, and number of children

Model 3 was adjusted for variables in Model 2 plus BMI, physical activity, calorie intake, smoking, alcohol drinking, tea drinking, family history of liver cancer, medical history of hepatitis, cholelithiasis, diabetes, and hypertension

The results of sensitivity presented that when excluding participants whose follow-up times were less than two years, the HRs and 95% CIs were similar to those of the main analyses at all follow-up times (Table 4).

**Table 4:** Sensitivity analysis: HRs and 95% CIs of liver cancer for residential mobility excluding participants with less than two years of follow-up (SWHS, 1996–2016)

	Cases (*n)*	Total (*n*)	PYs	HR (95% CI)1	HR (95% CI)^1^	HR (95% CI)^2^
Migration status						
No	189	63,431	1,113,720.6	1 (reference)	1 (reference)	1 (reference)
Yes	39	9,033	155,640.2	1.44 (1.41, 1.48)	1.47 (1.44, 1.50)	1.47 (1.44, 1.50)
Migration frequency						
No migration	189	63,431	1,113,720.6	1 (reference)	1 (reference)	1 (reference)
Once	31	6,508	111,776.3	1.49 (1.45, 1.53)	1.51 (1.48, 1.55)	1.51 (1.48, 1.55)
Twice or more times	8	2,525	43,863.9	1.34 (1.29, 1.40)	1.37 (1.32, 1.43)	1.37 (1.32, 1.43)
*P* for trend				< 0.001	< 0.001	< 0.001
Age at initial migration						
No migration	189	63,431	1,113,720.6	1 (reference)	1 (reference)	1 (reference)
Under 18 years old	8	2,091	35,078.3	1.85 (1.77, 1.94)	1.77 (1.69, 1.85)	1.76 (1.68, 1.84)
18–24 years old	11	2,254	38,141.1	1.69 (1.62, 1.76)	1.63 (1.56, 1.70)	1.62 (1.55, 1.69)
25–34 years old	11	2,757	48,610.0	1.29 (1.24, 1.34)	1.36 (1.31, 1.42)	1.36 (1.31, 1.42)
35 years old and above	9	1,931	33,810.8	1.18 (1.12, 1.23)	1.25 (1.19, 1.31)	1.26 (1.20, 1.32)
*P* for trend				< 0.001	< 0.001	< 0.001
Immigration to Shanghai status						
No	189	63,459	1,114,213.8	1 (reference)	1 (reference)	1 (reference)
Yes	39	9,005	155,147.0	1.44 (1.41, 1.48)	1.47 (1.44, 1.50)	1.47 (1.44, 1.50)
Age at first immigration to Shanghai						
No immigration	189	63,459	1,114,213.8	1 (reference)	1 (reference)	1 (reference)
Under 18 years old	6	1,274	21,004.0	2.29 (2.16, 2.42)	2.08 (1.97, 2.20)	2.06 (1.95, 2.18)
18–24 years old	8	1,646	26,977.6	2.16 (2.06, 2.27)	1.93 (1.83, 2.03)	1.91 (1.81, 2.00)
25–34 years old	10	3,011	53,136.0	1.33 (1.28, 1.38)	1.39 (1.34, 1.45)	1.39 (1.34, 1.44)
35 years old and above	15	3,074	54,029.5	1.17 (1.12, 1.21)	1.23 (1.19, 1.28)	1.24 (1.20, 1.29)
*P* for trend				< 0.001	< 0.001	< 0.001

Model 1 was unadjusted;

Model 2 was adjusted for age at entry, menopausal status, education, family income, occupation, marital status, and number of children

Model 3 was adjusted for variables in Model 2 plus BMI, physical activity, calorie intake, smoking, alcohol drinking, tea drinking, family history of liver cancer, medical history of hepatitis, cholelithiasis, diabetes, and hypertension

## Discussion

Although the association between residential mobility and cancer has raised a lot of concentration, the long-term influence of residential history remains underexplored. Our study examined the association between residential mobility and the risk of liver cancer among Chinese women and showed that domestic migration, especially immigration to Shanghai had positive associations with liver cancer risk. In addition, domestic migration frequency, age at initial migration and first immigration to Shanghai, had linear trends with an elevated liver cancer risk. The results remained stable after adjusting different confounding factors or excluding participants with less than two years of follow-up.

Migration is an important social phenomenon [8]. China, the world’s most populous country, has experienced significant domestic migration over the past century [9]. However, the association of domestic migration with health remains unclear. On the one hand, migration has positive effects on health. A cross-sectional study indicated a positive impact of short-term migration on health when the economic conditions improved (estimate = 0.067, *P* = 0.004) [20]. Moreover, in Indonesia, many migrants live in areas that do not provide piped water (OR = 6.41, 95%CI, 3.97–10.36) and flush toilets (OR = 3.44, 95%CI, 2.10–5.65) than rural stayers, which had a positive effect on their health [21]. On the other hand, migration had a negative influence on several pathways to health. Firstly, migration could expose migrants to mental disorders triggered by less social support and family separation. A case-control study found that domestic migration had a positive association with depressive symptoms (OR = 1.07, 95% CI, 1.02–1.12), driven by parent-child interaction (4.45%, *P* < 0.01) [9]. Secondly, migrants usually work for a long time and face heavy working stress in unfavorable environments, which puts migrants at a higher risk of disease. A cross-sectional study showed that rural-to-urban migrants lacking skills tend to be employed in dangerous and dirty jobs, which induced health problems [22]. Finally, many migrants lack sufficient access to health services, which is harmful to their health. Domestic migration experience was found to be associated with negative associations on health (estimate = 0.082, *P* = 0.039), and failure to seek medical treatment when necessary (17.92%, *P* = 0.006) was shown to mediate this association [8]. In our study, the positive association of domestic migration on liver cancer risk could share similar pathways of domestic migration on negative health effects, which needs more information about the potential mediating variables in further studies.

In addition, residential history enables evaluation of the influence on environmental exposure risks. Previous long-term studies have identified several risk factors for liver cancer, such as hepatitis B virus (HBV) infection, aflatoxin-exposed foods, and contaminated water, especially in high-risk areas [15, 23, 24]. A meta-analysis indicated that Chinese rural areas had a higher prevalence of HBV infection than urban areas, and the highest prevalence of HBV infection was reported in Western provinces, which could lead to a high incidence of liver cancer [24]. A cross-sectional study in China evaluated the aflatoxin exposure levels of the local residents living in Guangxi Zhuang Autonomous Region and Jiangsu Province, and found that these people with high HCC incidence all had high levels of aflatoxin exposure [15]. A review concluded that the risk factors in drinking water, especially blue-green algal toxins, are cancer-promoting agents for hepatitis and HCC, especially in Jiangsu Province, China [23]. Our study found that compared with those living in Shanghai for the whole time, women who immigrated to Shanghai had a higher risk of liver cancer occurrence, which could be attributed to greater opportunities for migrants to experience adverse environmental exposures. Detailed information about HBV infection, aflatoxin-exposed food intake, and contaminated water is needed for further research.

As for migration frequency, many previous studies have shown that having more experiences of domestic migration could be associated with an increased health risk, due to new environmental stressors [8, 9]. Whereas in our study, the high risk of liver cancer was slightly reduced when compared to the migration for two or more times with once. The reason might be attributed to those people who have migrated for more time having adapted to the environment of frequent migration and made the advantages of migration outweigh the disadvantages, and reducing the risk of adverse health outcomes. However, the cases of migrated for more times in our study are small, and longer follow-up times are needed to identify the association of migration frequency with liver cancer risk.

Age at initial migration is also important in reflecting the effect of residential mobility on lifetime health. Our study found that those who were younger at first domestic migration, especially immigration to Shanghai, had a higher risk of liver cancer incidence, while older individuals had a relatively lower risk. A case-control study found that a stable childhood environment was associated with a significantly increased rate of self-reported health later in life (OR = 1.42, 95% CI, 1.18–1.72) [14]. In contrast, child migrants are more vulnerable to negative effects. Many studies have identified the factors associated with children’s residential mobility and negative health status, including adverse environmental exposures, higher levels of behavioral problems, adolescent depression, and reduced access to healthcare [25–27]. Older people were more likely to receive social welfare and sufficient healthcare, which could mitigate the negative effects on their health status [8, 9].

Previous relevant studies are mainly cross-sectional studies [10, 13] or case-control studies [11, 12], which analyze the retrospective information and couldn’t get a reliable causal relationship, and many of the studies focus on more extensive health problems like chronic diseases and depressive symptoms [8, 9]. Internationally, our study is the first cohort study to investigate the relationship between residential mobility on liver cancer incidence among Chinese women. Our study is a prospective study, and the study design is a population-based cohort. We recruited participants living in Shanghai, one of the largest immigration cities in the mainland of China over the past few decades, to analyze the most representative immigration behavior that affects health. Furthermore, we adjusted the confounding factors identified in previous publications and our studies, which can eliminate the potential adverse influence of potential bias and present the exact effect of residential mobility on liver cancer risk. However, some limitations still need to be addressed. Firstly, we only obtained residential histories from self-reported surveys at baseline, which might induce recalling bias and no information alterations during follow-up, so the findings need to be interpreted with caution. Secondly, the data on potential mediating variables was incomplete and limited, such as the detailed information about contaminated water, aflatoxin-exposed food intake, welfare attainment, and medical service utilization, which need to be considered in further studies. Thirdly, data on HBV or HCV infection assays were lacking, while the medical history of chronic hepatitis was adjusted to reduce the problem. In addition, the number of migration cases is small, and longer follow-up times are needed to identify the association of residential mobility with liver cancer risk. Finally, as our prospective cohort primarily consisted of urban Chinese adult women, it should be cautious to generalize the findings to a rural or male population.

## Conclusion

In conclusion, our study indicated a possible association between residential mobility and an increased risk of liver cancer among Chinese women, highlighting the adverse role of domestic migration on liver cancer development. It is needed to implement effective interventions to reduce the cumulative adverse effects of residential mobility and improve people’s health. Further collection of epidemiological evidence and evaluation of the mediating variables are still needed for further studies.

## Data Availability

The data will be available on request pending approval by the scientific committee of the relevant institutes.

## Abbreviations

HBV: Hepatitis B virus
HCV: Hepatitis C virus
SWHS: Shanghai women’s health study
BMI: Body mass index
MET: Metabolic equivalent
HR: Hazard ratio
CI: Confidence interval
PYs: Person-years
